# Round the Hospitals

**Published:** 1920-10-30

**Authors:** 


### ROUND THE HOSPITALS.

The Extraordinary General Meeting of the College of Nursing, to be held on Thursday, November 4, at 4 p.m., at the Royal Society of Medicine, 1 Wimpole Street, W. 1, will bring a large influx of members to London and test the accommodation at 7 Henrietta Street to the uttermost. The first resolution to be submitted relates to the suggested annual subscription, which is to be fixed by the Council and may not exceed twenty shillings. Life membership, it appears, is to be reckoned at the rate of twenty annual subscriptions, and half may be paid in yearly instalments. For instance, a member, after paying an annual subscription for ten years, may pay in a lump sum the amount for ten more years and become a life member. The second resolution is intended to safeguard the interests of members "with regard to whom an inquiry is to be held," and give them the "opportunity of stating their case and defending themselves before the Council." A second extraordinary general meeting has to be held on November 20 with a view to obtaining confirmation of the special resolutions, should they be carried by the requisite majority (see page vi).

We have received some highly interesting statistics from Miss Brown, Matron of the Royal Victoria Infirmary, Newcastle-on-Tyne, bearing on the vexed question of the proportion of probationers who break down under training. The hospital was opened in October 1906, and the figures are given from that date till October in this year. During these fourteen years the number of probationers who signed the agreement with the hospital was 520. At first, until 1911, the agreement was for three years' training; after that year the term was extended to four years. The number of probationers who broke down during training within this period was seventeen, excluding the case of two who died during the severe influenza epidemic last year. The percentage of failures on account of illness is therefore 3.2. Thirteen of these occurred in the second year, three in the first, and but one in the fourth year. The disabilities which led to abandonment of training were various. There were three cases of gastric trouble, one of which had been a former malady, concealed by the probationer on entrance. There were three cases of tubercular disease, one following an attack of measles with pneumonia. General debility and debility following appendicitis account for two more. Then there are such complaints as psoriasis, eczema of hands, rheumatism, functional tremor, deafness. One case of Bright's disease and two of anæmia finish the list. Inquiries made some years ago led us to believe that the percentage of probationers who broke down in good training-schools did not exceed five per cent. We should welcome further statistics extending over a term of years as a contribution to this question, too often obscured by ignorance or prejudice.

Suggestions of the medical officer of health for the amendment of the existing code of rules of the C.M.B. were further considered at the last meeting of the Plymouth Health Committee. A letter was read from the South Devon and South-East Cornwall Midwives' Institute objecting to compulsory notification of all bookings of midwives. The Committee agreed to submit the following rules to the C.M.B. for adoption: (1) No midwife should be permitted to certify a still birth unless actually present at the birth; (2) pupils should be forbidden to attend confinements unless in the presence of a certificated midwife; and (3) miscarriages and abortions should be notified as in still-births.

The rather pessimistic speeches at the recent Tuberculosis Conference have brought forcibly before the public the breakdown in sanatorium treatment, round which high hopes have centred. The return home of the convalescent patient proves in far too many cases a prelude to steady deterioration, and until some means can be found of counteracting the effect of the old associations, inducing a return of former symptoms, progress along these lines would seem to be barred. The mental aspect of tuberculosis has been too little studied, although there are striking facts which illustrate the extent to which the mind or imagination can raise or reduce resistance to the disease. So far almost the only means of stimulating mental resistance is to be found in the personality of the doctors and nurses engaged in combating tuberculosis in sanatoria or other centres. The problem is to maintain resistance after the return to normal conditions, and this has never been systematically carried out. The task of removing to special colonies all persons affected by the disease is too stupendous to be worth talking about; but under a well-planned scheme, with skilled nurses co-operating, it should be possible for the convalescents to be kept in good heart after their return home and helped over the couple of years which intervene between apparent and real cure. The call is for good organisation.

The annual general meeting of the Ladies' Guild of St. Thomas's will be held this week. A record attendance is desired, for the needs to which the Guild ministers have never been more pressing, and the departments to which it relates are all hard pressed for money. New workers and fresh subscribers are urgently wanted to take the place of some whose labours are now at an end.

Affiliation is gaining ground. The Devonport Guardians have adopted the report of their Infirmary Committee, under which an arrangement is projected with the Royal Albert Hospital, whereby the nurses of the Poor-law Infirmary can have twelve months' training in the hospital and thus be in a position to qualify for the final nursing examination. There is no resident medical officer in the

**ROUND THE HOSPITALS—*(continued)*.**

infirmary, so that the probationers are at a disadvantage. The Board has agreed to pay the nurses' salaries and bonus while they are at the hospital, and at the end of their twelve months' training they will be asked to give a full year's service to their former training-school.

---

Loughborough is one of the places where the management of a nursing association is well understood. At the annual meeting of the Queen's Nursing Association in the city, at which the Mayor presided, the report read testified to a great growth in the work which would shortly entail the addition of another nurse to the staff, and a great access of public support. One collector during the year had obtained 175 new subscriptions. Over £20 was received from the patients, whose appreciation is warm and constant.

---

The incapable and extravagant method adopted by Poor-law authorities in feeding the old people committed to their charge is the subject of severe strictures by Miss Edith Sellers in the October number of the *Nineteenth Century*. Miss Sellers has visited (before the war) old-age homes in this country and in rural Sweden, Denmark, Lapland, and Switzerland, (in urban Norway, Germany, Austria, and Russia; in Roumania, in Serbia, in Bosnia, and she finds in all these homes better conditions at a far lower cost than prevail in London workhouses. The cost per head of workhouse inmates has risen to fabulous proportions. "In one London workhouse last year it was £65; in another £77; in another again £95 11s." Will the time never come when business management and humane methods will be combined and our old people be rendered cheerful and content in the last years of their lives?

---

The American Red Cross have taken energetic steps to counter the danger of infection by typhus, which attends the repatriation of 300,000 Letts from Russia under the recent agreement with the Lithuanian Government. Five hundred out of the number, men, women, and children have recently crossed the Russian frontier, and were met at the Reschitza station by the Red Cross Commission, and passed into a quarantine station. All were found to be swarming with vermin, so that the first thing to be done is to give hot baths with special treatment, and provide fresh clothing. They are kept in quarantine for five days, during which good food is provided and beds in warm dormitories. Hospital treatment is provided for such as are ill. This elaborate system of disinfection is necessary to prevent the country from being devastated with typhus. The quarantine buildings cover an extent of a square mile and entail a large service of doctors, nurses, and assistants. Similar stations are required at all the main crossing places where refugees and returned prisoners issue from Russia, and the League of Red Cross Societies can establish them if sufficient means are forthcoming. Subscriptions may be sent to the Imperial War Relief Fund, Fishmongers' Hall, E.C. 4.

---

The most effective ways of meeting the increased cost of bread are by eating less of it and by baking at home. This is far cheaper, and, if the right flour be used, doubly economical, as a smaller amount is required. For full information concerning bread, its place in the diet of children and of adults, advice on the selection of flour for bread, scones and cakes, and how to obtain practical lessons, public or private, in baking or cookery, apply, enclosing stamped addressed envelope, to the Food Education Society, Danes Inn House, 265 Strand, London, W.C. 2.

---

Acting upon the authority of the Court of Governors, the House Committee of Guy's Hospital have decided to raise the salaries of probationers to £20, £25, £30 for first, second, and third year's training respectively.

---

A novel idea was put forward by Mr. Gillett when presiding at the opening of a sale of work recently held at Walthamstow in aid of the Hospital Linen League, when he suggested the benefit that would accrue to the institution by the formation of a "potato league," by which the men of the district would undertake to provide the hospital with a regular supply of vegetables, etc., as was done in the case of linen by the ladies. In connection with the latter work it is interesting and satisfactory to record what has been done by the members: started in 1912 with a capital of £45, the League provided 364 articles that year, and for the next six (1913-1918) kept the hospital stock complete and replenished as occasion required; towards the end of 1918, however, greater needs arose, and as the result of special efforts 191 articles were made then and 232 in the following year, while to provide for the present needs the sale of work already referred to was held, the proceeds of which amounted to over £60.

---

Southwark Board of Guardians were informed by their Infirmary Visiting Committee that there had been frequent falls of ceilings in various rooms at their infirmary. A separation ward, which had been converted into an operating-room, was being used for an abdominal operation upon a woman patient, who was still lying unconscious upon the table, when some of the ceiling fell. Fortunately the patient was not injured, and the only one actually struck was Dr. Bruce, the Medical Superintendent, who stated that if it had occurred a few minutes earlier very serious, and probably fatal, consequences might have resulted. The committee gave urgent instructions that the ceiling should at once be replaced by composition slabs. The infirmary for some time has been used as a military hospital, and the possibility is that the ceilings and other structural defects have not been watched with the same keenness as they are when the guardians have entire control.

